# Corticosteroids Increase the Risk of Invasive Fungal Infections More Than Tumor Necrosis Factor-Alpha Inhibitors in Patients With Inflammatory Bowel Disease

**DOI:** 10.1093/crocol/otad010

**Published:** 2023-02-19

**Authors:** Martin H Gregory, Andrej Spec, Dustin Stwalley, Anas Gremida, Carlos Mejia-Chew, Katelin B Nickel, Matthew A Ciorba, Richard P Rood, Margaret A Olsen, Parakkal Deepak

**Affiliations:** Inflammatory Bowel Diseases Center, Division of Gastroenterology, Washington University School of Medicine, Saint Louis, Missouri, USA; Division of Infectious Diseases, Department of Medicine, Washington University School of Medicine, Saint Louis, Missouri, USA; Institute for Informatics, Washington University School of Medicine, Saint Louis, Missouri, USA; Inflammatory Bowel Diseases Center, Division of Gastroenterology, Washington University School of Medicine, Saint Louis, Missouri, USA; Division of Infectious Diseases, Department of Medicine, Washington University School of Medicine, Saint Louis, Missouri, USA; Division of Infectious Diseases, Department of Medicine, Washington University School of Medicine, Saint Louis, Missouri, USA; Inflammatory Bowel Diseases Center, Division of Gastroenterology, Washington University School of Medicine, Saint Louis, Missouri, USA; Inflammatory Bowel Diseases Center, Division of Gastroenterology, Washington University School of Medicine, Saint Louis, Missouri, USA; Division of Infectious Diseases, Department of Medicine, Washington University School of Medicine, Saint Louis, Missouri, USA; Inflammatory Bowel Diseases Center, Division of Gastroenterology, Washington University School of Medicine, Saint Louis, Missouri, USA

**Keywords:** Crohn’s disease, ulcerative colitis, candidiasis, histoplasmosis, immune suppression

## Abstract

**Background:**

Invasive fungal infections are a devastating complication of inflammatory bowel disease (IBD) treatment. We aimed to determine the incidence of fungal infections in IBD patients and examine the risk with tumor necrosis factor-alpha inhibitors (anti-TNF) compared with corticosteroids.

**Methods:**

In a retrospective cohort study using the IBM MarketScan Commercial Database we identified US patients with IBD and at least 6 months enrollment from 2006 to 2018. The primary outcome was a composite of invasive fungal infections, identified by ICD-9/10-CM codes plus antifungal treatment. Tuberculosis (TB) infections were a secondary outcome, with infections presented as cases/100 000 person-years (PY). A proportional hazards model was used to determine the association of IBD medications (as time-dependent variables) and invasive fungal infections, controlling for comorbidities and IBD severity.

**Results:**

Among 652 920 patients with IBD, the rate of invasive fungal infections was 47.9 cases per 100 000 PY (95% CI 44.7–51.4), which was more than double the TB rate (22 cases [CI 20–24], per 100 000 PY). Histoplasmosis was the most common invasive fungal infection (12.0 cases [CI 10.4–13.8] per 100 000 PY). After controlling for comorbidities and IBD severity, corticosteroids (hazard ratio [HR] 5.4; CI 4.6–6.2) and anti-TNFs (HR 1.6; CI 1.3–2.1) were associated with invasive fungal infections.

**Conclusions:**

Invasive fungal infections are more common than TB in patients with IBD. The risk of invasive fungal infections with corticosteroids is more than double that of anti-TNFs. Minimizing corticosteroid use in IBD patients may decrease the risk of fungal infections.

## Introduction

Inflammatory bowel disease (IBD) is characterized by pathologic inflammation in the gastrointestinal tract, so logically immunosuppression is the primary treatment modality.^1^ Tumor necrosis factor-alpha inhibitors (anti-TNFs) are more selective immunosuppressants than corticosteroids and are associated with a lower risk of bacterial infection.^2,3^ However, the anti-TNF mechanism of action may make patients especially susceptible to invasive fungal infections, a potentially devastating complications of IBD treatment.^4^

TNF is essential for granuloma formation, which is needed for defending against fungal infections and tuberculosis (TB).^4^ Reactivation of latent TB is a well-described side effect of anti-TNF therapy.^5^ In addition, reports of invasive fungal infections, when anti-TNFs became available, led to a black box warning for anti-TNFs.^4^ However, there have been no large studies examining the risk of specific invasive fungal infections in patients with IBD, particularly among those treated with anti-TNFs compared with corticosteroids. With the rise in the incidence of IBD and increasing use of anti-TNFs,^6^ clarifying the risks of complications with different IBD treatments is essential to appropriately weigh the risks of treatment for individual patients.

Our aims were to identify how common invasive fungal infections are among IBD patients in the United States and to identify risk factors for invasive fungal infections. For reference, we compared the incidence to both latent TB and TB disease, which all IBD patients should be screened for prior to initiating biologic therapy. We hypothesized that invasive fungal infections are much more common than TB in IBD patients in the United States. Our second aim was to identify risk factors for invasive fungal infections, specifically focused on IBD medications. Despite a mechanism of action that may predispose patients on anti-TNFs to fungal infections, we hypothesized that corticosteroids are associated with higher risk of invasive fungal infections than anti-TNFs.

## Materials and Methods

This was a retrospective cohort study using the IBM MarketScan Commercial Database from 2006 to 2018. This database includes inpatient, outpatient, and pharmacy claims from participating private insurance health plans and large employers throughout the United States. It contains data from more than 150 million employees and their dependents. It includes a variety of payment models, including fee-for-service, preferred provider organizations and capitated health plans.

We used a previously validated algorithm designed to identify patients with IBD by International Classification of Diseases (ICD) 9 coding.^7^ Patients were required to have at least 2 ICD-9/10-CM diagnosis codes for Crohn’s disease (CD) or ulcerative colitis (UC), with at least 1 coded on an outpatient visit. Patients were required to have at least 6 months of health insurance enrollment before infection to identify comorbidities. We did not exclude patients by age, but since the IBM MarketScan Commercial Database does not include Medicare plans, all patients were younger than 65 years.

The primary outcome was a composite of invasive fungal infections, including invasive candidiasis, histoplasmosis, coccidiomycosis, aspergillus, cryptococcus, *Pneumocystis jiroveci*, blastomycosis, mucormycosis, paracoccidiomycosis, fungal pneumonia, and fungal meningitis. Since there has been no validated definition for fungal infection using administrative data, we required all patients coded for one of the invasive fungal infections to fill a prescription for an antifungal medication within 30 days of a fungal diagnosis code to reduce the likelihood of coding error (eg, history of previously treated infection, unconfirmed infection). Diagnosis codes for *P. jiroveci* were required to have a prescription for one of the following within 30 days (trimethoprim–sulfamethoxazole, atovaquone, clindamycin and primaquine, trimethoprim and dapsone, or pentamidine). Only the first instance of fungal infection was counted to avoid identifying subsequent coding for the same infection as a new infection (see Supplementary Table S1 for list of diagnosis and procedure codes used). Since esophageal candidiasis is less severe than invasive candida infections, it was not included in the primary analysis.

TB infection was a secondary outcome. We defined TB disease as an ICD-9/10-CM diagnosis code for active TB and prescriptions for isoniazid plus 2 other medications (one of rifampin, rifapentine, or rifabutin, and one of ethambutol, pyrazinamide, or moxifloxacin) within 30 days. Latent TB was defined as an ICD-9/10-CM diagnosis code for a positive TB skin or blood test with a prescription for isoniazid or rifampin alone for at least 90 days. Patients with a code for TB disease and a prescription for only isoniazid or rifampin were considered as latent TB. Additional secondary outcomes were individual fungal infections.

Baseline comorbidities, IBD medications, emergency department visits, hospitalizations, IBD-related surgeries, and opioid prescriptions were captured in the first 6 months of enrollment (see Supplementary Table S1 for list of diagnosis and procedure codes used). The severity of CD was captured using codes for complicated CD, either CD-related fistula or stricture during follow-up. For UC, we captured disease location (proctitis, left-sided colitis, and pancolitis).

### Statistical Analysis

The incidence rate for the primary outcome was calculated by counting the number of persons with invasive fungal infections divided by the sum of patient follow-up time, expressed as a rate per 100 000 person-years (PY). CIs were estimated with Poisson regression. The incidence rate of TB disease and latent TB infection was calculated similarly.

A Cox proportional hazards model using time-dependent variables for IBD medications was used to determine the association of IBD medications with invasive fungal infection. Patients were censored when they lost health insurance coverage, died, or at the end of the study period (12/31/2018). Follow-up time for each patient was divided into 90-day intervals. We determined whether a patient had a prescription for an IBD medication during each interval. Thus, the association between an IBD medication and a fungal infection was only assessed while the patient was on that medication. We chose a 90-day interval because several medications of interest are administered every 60 days, and a 90-day window would account for scheduling delays (eg, insurance authorization). We tested 30- and 60-day treatment intervals in sensitivity analyses. The primary medications of interest were anti-TNFs (adalimumab, infliximab, certolizumab pegol, and golimumab) and oral corticosteroids. In a separate analysis we examined immunomodulators (azathioprine and methotrexate) as time-dependent variables, both alone and in combination with anti-TNFs and corticosteroids. For each 90-day interval, we determined if a patient had a prescription for a corticosteroid, an immunomodulator or an anti-TNF and whether an invasive fungal infection occurred. Mutually exclusive variables were created for each combination of the 3 medications. Using no medication as the reference, we assessed the hazard ratio (HR) for time to invasive fungal infection for each combination of the time-dependent variables. Since vedolizumab (5/20/2014) and ustekinumab (9/23/2016) were approved later in the follow-up period, there were too few fungal infections to assess the risk with these medications. Since there is evidence that even low-dose or short-term corticosteroid use is associated with increased risk of infection, we included any oral steroid prescription (prednisone, dexamethasone, prednisolone, and budesonide) in the 90-day treatment period as being on corticosteroids.^8,9^ In sensitivity analysis we included only moderate or high-dose steroids, defined as 20 mg of prednisone or more for at least 14 days. For the sensitivity analysis, budesonide was not included as steroid treatment. Since esophageal candidiasis is less severe than other invasive candida infections, we excluded esophageal candidiasis from the primary analysis but did a separate sensitivity analysis including it in the primary fungal infection outcome.

In the multivariate Cox proportional hazards model, we controlled for IBD disease severity by including variables found to be associated with IBD-related hospitalization in administrative research.^10^ This publication used separate models for CD and UC. Since we combined UC and CD, we included variables that were found to be associated with IBD-related hospitalization in each of the models, including age, sex, anemia, opioid prescription, emergency room visit, hospitalization, or IBD-related surgery in the baseline period (see Supplementary Table S1 for list of diagnosis and procedure codes used).^10^ For CD severity we included fistulizing or stricturing CD and for UC we included disease location (pancolitis, left-sided colitis, and proctitis). Prescription for total parenteral nutrition (TPN) was included as a time-dependent variable in the model as TPN is a risk factor for fungemia.^11^ We assessed whether a patient had a procedure code for TPN during each 90-day interval (see Supplementary Table S1 for list of diagnosis and procedure codes used). We also controlled for the presence of diabetes mellitus, leukemia, and lymphoma during the baseline period, as these are known risk factors for fungal infection.^12,13^ SAS version 9.4 was used for all statistical analyses.

### Ethical Considerations

The Washington University Human Resources Protection Office exempted this study from oversight by the Institutional Review Board.

## Results

We identified 652 920 patients with IBD, including 353 165 with UC and 291 506 with CD (Table 1). There were 8225 patients with codes for both UC and Crohn’s. The median follow-up was 1.6 years (interquartile range: 2.8 years). Among Crohn’s patients, 13 075 (4.5%) had stricturing disease and 5817 (2.0%) had a fistula. The mean age was 42.2 years (range 0–65). The population was 54.6% female.

**Table 1.** Baseline characteristics of patients with inflammatory bowel disease in the IBM MarketScan database from 2006 to 2018.

Characteristic	*N* (%)
Total IBD	652 920 (100)
Ulcerative colitis	353 165 (54.09)
Pancolitis	274 190 (77.64)
Left-sided	37 346 (5.72%)
Proctitis	41 629 (6.38%)
Crohn’s disease	291 506 (44.65)
Stricturing	13 075 (4.49)
Penetrating	5817 (2.00)
IBD-unclassified	8225 (1.26)
Medications	
Aminosalicylates	304 722 (46.67)
Immunomodulator^a^	116 400 (17.83)
Corticosteroids	
Systemic	313 781 (48.06)
Budesonide	70 895 (10.86)
Anti-TNF	103 755 (15.89)
Ustekinumab	3597 (0.55)
Vedolizumab	8149 (1.25)
Female	356 769 (54.64)
Age	
<18	35 249 (5.40)
18–24	56 640 (8.67)
25–29	51 965 (7.96)
30–34	57 837 (8.86)
35–39	62 340 (9.55)
40–44	67 545 (10.35)
45–49	74 526 (11.41)
50–54	88 082 (13.49)
55–59	83 774 (12.83)
60+	74 962 (11.48)
Comorbidities	
Diabetes mellitus	23 737 (3.64)
Chronic kidney disease	2748 (0.42)
Hypertension	45 635 (6.99)
Anemia	22 779 (3.49)
Opioid prescription	140 496 (21.52)
Leukemia	485 (0.07)
Lymphoma	970 (0.15)
ED visit, hospitalization, or IBD surgery^b^	178 660 (27.36)
Total parenteral nutrition	3727 (0.57)
Follow-up in years after first code for IBD	
Mean	2.48
Minimum	0.003
25th percentile	0.64
Median	1.59
75th percentile	3.47
Maximum	12.50

Abbreviations: Anti-TNF, anti-tumor necrosis factor-alpha inhibitors; IBD, inflammatory bowel disease.

^a^Includes azathioprine and methotrexate.

^b^Included all-cause emergency department (ED) visits, all-cause hospitalizations, or IBD-related surgery. See Supplementary Table S1 for list of codes used.

Almost half (48.1%) of patients had a prescription for oral corticosteroids during follow-up. There were 103 755 patients (15.89%) who received a prescription for an anti-TNF at some point during follow-up. An immunomodulator was prescribed in 116 400 (17.8%) of patients. Ustekinumab and vedolizumab were prescribed in 3597 (0.55%) and 8149 (1.25%), respectively. There were 3727 (0.6%) patients who received TPN during follow-up.

**Figure 1.**
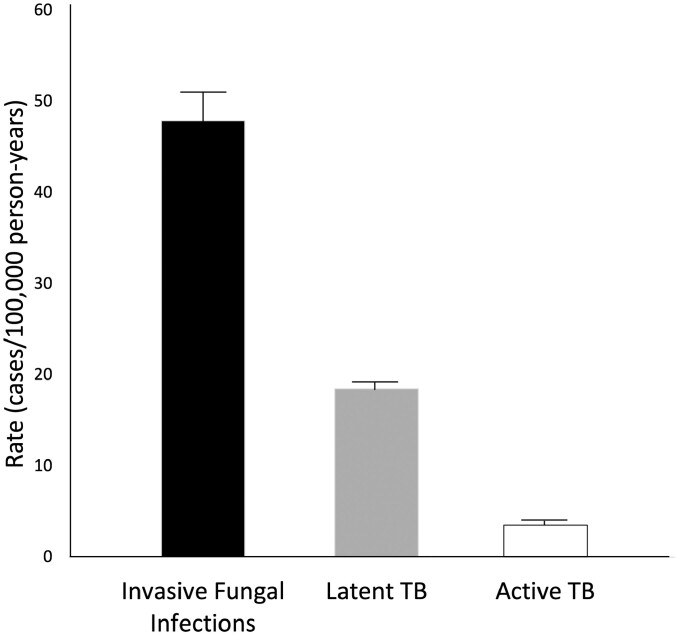
Incidence of invasive fungal infections and tuberculosis in patients with inflammatory bowel disease.

There were 775 fungal infections during 1 616 941 years of follow-up for a rate of 47.9 per 100 000 PY (95% CI 44.7–51.4) (Figure 1 and Table 2). Histoplasmosis was the most common invasive fungal infection (12 per 100 000 PY [95% CI 10.4–13.8]), followed by candidiasis (9.3 per 100 000 PY [95% CI 7.9–10.9]) and coccidiomycosis (8.8 per 100 000 PY [95% CI 7.4–10.3] (Table 2) Other fungal infections were rare. There were 305 cases of latent TB (18.5 cases per 100 000 PY [95% CI 16.6–20.8]) and 60 cases of TB disease (3.5 cases per 100 000 PY [95% CI 2.7–4.6]) (Figure 1).

**Table 2.** Organisms causing invasive fungal infections in patients with inflammatory bowel disease.

	Cases (*N*, %)	Rate (cases/100 000 person-years)	95% lower CI	95% upper CI
Invasive fungal infection	775 (100)	47.93	44.67	51.42
Histoplasmosis	194 (25.03)	11.99	10.41	13.80
Invasive candidiasis	150 (19.35)	9.27	7.90	10.88
Coccidiomycosis	142 (18.32)	8.77	7.44	10.34
Aspergillus	119 (15.35)	7.35	6.14	8.80
* Pneumocystis jiroveci*	81 (10.45)	5.00	4.03	6.22
Cryptococcus	46 (5.94)	2.84	2.13	3.79
Blastomycosis	12 (1.55)	0.74	0.42	1.31
Fungal pneumonia	20 (2.58)	1.24	0.79	1.92
Mucormycosis	6 (0.77)	0.37	0.17	0.83
Fungal meningitis	3 (0.39)	0.19	0.06	0.57
Paracoccidiomycosis	2 (0.26)	0.12	0.03	0.49

**Figure 2.**
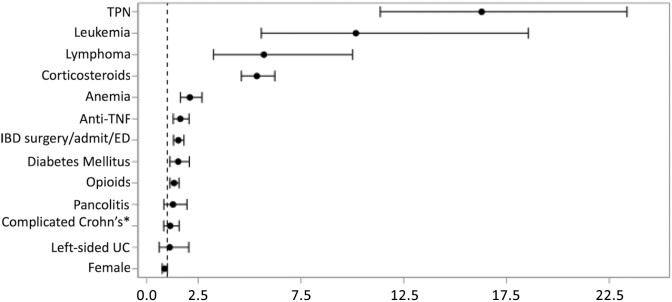
Hazard ratios from a Cox proportional hazards model for risk of fungal infections in patients with inflammatory bowel disease. Abbreviations: Anti-TNF, anti-tumor necrosis factor-alpha inhibitors; IBD, inflammatory bowel disease; TPN, total parenteral nutrition. Anti-TNF, corticosteroids, and TPN were time-dependent variables. Model also adjusted for age. See Supplementary Table S2 for hazard ratios for age groups. *Includes IBD-related surgery, all-cause emergency department (ED) visits, and all-cause hospitalizations in the baseline period. See Supplementary Table S1 for list of codes used. **Includes Crohn’s-related fistula or stricture. See Supplementary Table S1 for list of codes used.

After controlling for comorbidities, IBD severity and TPN use, both corticosteroids (HR 5.4, CI 4.6–6.2) and anti-TNF use (HR 1.6, CI 1.3–2.1) were associated with increased risk of invasive fungal infections (Figure 2 and Supplementary Table S2). TPN was associated with a 16-fold increase in the risk of invasive fungal infection (CI 11.4–23.4). Anemia, diabetes mellitus, leukemia, and lymphoma were also associated with significantly increased risk of fungal infection. In sensitivity analysis, using 30- or 60-day intervals for medication exposure instead of 90-day intervals, had minimal effect on the HRs for anti-TNFs and corticosteroids (see Supplementary Tables S3 and S4). In an additional sensitivity analysis limiting corticosteroid exposure to only moderate or high dose corticosteroids (see definition in methods), the results were similar (see Supplementary Table S5). Results were similar if esophageal candidiasis was included in the primary outcome (see Supplementary Table S6). Immunomodulators alone were not associated with an increased risk of invasive fungal infection (HR 1.1, CI 0.8–1.6) and the risk of invasive fungal infection with immunomodulator and anti-TNF combination therapy (HR 1.6, CI 0.7–3.7) was similar to anti-TNF monotherapy (HR 2.5, CI 1.7–3.7) (Table 3). The addition of corticosteroids to any regimen significantly increased the risk of invasive fungal infection. For example, the addition of corticosteroids to anti-TNF increased the HR from 2.5 (CI 1.7–3.7) to 8.1 (CI 5.7–11.6).

**Table 3.** Hazard ratios from a Cox proportional hazards model for risk of invasive fungal infections in patients with inflammatory bowel disease including immunomodulators.

	Hazard ratio	95% lower	95% upper	*P*
Anti-TNF monotherapy^a^	2.486	1.691	3.653	<.0001
Corticosteroids only^a^	5.655	4.764	6.714	<.0001
Immunomodulator monotherapy^a^^,^^b^	1.099	0.749	1.614	.6294
Anti-TNF + immunomodulator^a^^,^^b^	1.648	0.733	3.704	.2268
Immunomodulator + corticosteroids	6.012	4.550	7.943	<.0001
Anti-TNF + corticosteroids^a^^,^^b^	8.100	5.679	11.551	<.0001
Anti-TNF + immunomodulator + corticosteroids^a^^,^^b^	6.628	3.790	11.591	<.0001
TPN	16.467	11.483	23.614	<.0001
Female	0.871	0.751	1.010	.668
Diabetes mellitus	1.529	1.127	2.074	.0063
Complicated Crohn’s^c^	1.136	0.820	1.572	.4438
UC disease location				
Ulcerative proctitis	Ref			
Ulcerative left-sided	1.107	0.604	2.030	.7414
Pancolitis	1.260	0.822	1.931	.2890
IBD surgery/admit/ED^d^	1.539	1.304	1.817	<.0001
Anemia	2.088	1.627	2.680	<.0001
Opioids	1.330	1.127	1.589	.0008
Leukemia	10.124	5.549	18.474	<.0001
Lymphoma	5.694	3.238	10.014	<.0001
Age				
<18	1.171	0.751	1.824	.4866
18–24	Ref			
25–29	1.120	0.718	1.746	.6182
30–34	1.275	0.846	1.921	.2449
35–39	1.216	0.810	1.826	.3449
40–44	1.431	0.972	2.107	.0697
45–49	1.660	1.145	2.406	.0075
50–54	1.541	1.065	2.228	.0217
55–59	1.677	1.160	2.426	.0060
60–64	1.771	1.193	2.631	.0046

Abbreviations: Anti-TNF, anti-tumor necrosis factor-alpha inhibitors; IBD, inflammatory bowel disease; TPN, total parenteral nutrition; UC, ulcerative colitis.

^a^Time-dependent variables.

^b^Immunomodulator includes azathioprine or methotrexate.

^c^Includes Crohn’s-related fistula or stricture. See Supplementary Table S1 for list of codes used.

^d^Includes IBD-related surgery, all-cause emergency department (ED) visits, and all-cause hospitalizations in the baseline period. See Supplementary Table S1 for list of codes used.

## Discussion

In this study, invasive fungal infections were 2 times more common than TB in patients with IBD. While both corticosteroids and anti-TNFs were associated with increased risk of invasive fungal infection, the risk with corticosteroids was more than 3-fold higher than with anti-TNFs alone or in combination with an immunomodulator. Additionally, TPN was associated with an 16-fold increased risk of invasive fungal infection.

To our knowledge, no large studies have examined the risk of fungal infections specifically in IBD patients. A smaller prospective cohort study of patients on anti-TNFs for multiple indications in France found an age- and sex-adjusted incidence rate for all non-TB opportunistic infections of 151.6 per 100 000 PY.^14^ Similar to our study, both anti-TNFs and corticosteroids were associated with increased risk of opportunistic infection.^14^ Another study of non-viral opportunistic infections found patients on anti-TNFs had an infection rate of 270 per 100 000 PY.^15^ This rate was higher than in our study but only 40% of the opportunistic infections were fungal, and the study included older patients and those on anti-TNFs for other immune-mediated inflammatory diseases.^15^ Similar to our study, both anti-TNF and corticosteroids were associated with increased risk of opportunistic infection, with corticosteroids associated with higher risk.^15^

Anti-TNFs have a black box warning for invasive fungal infection based on case reports.^16^ Indeed, we found that IBD patients on anti-TNFs were at increased risk for invasive fungal infections. However, in our study corticosteroid exposure was associated with a much greater risk of fungal infection than anti-TNF. Thus, our results suggest anti-TNFs should be preferred over continued exposure to corticosteroids, with respect to the risk of fungal infections. It is possible that ustekinumab or vedolizumab may be a good alternative for IBD patients at a high risk for invasive fungal infection, such as those on TPN or hematologic malignancy. Unfortunately, since these agents were approved at the end of our follow-up period, we had too few patients on these therapies to assess the risk of invasive fungal infections with these newer therapies. Future studies should examine whether these agents are associated with a lower risk of invasive fungal infections than anti-TNFs.

Given the rarity of TB, it may be reasonable to forego annual TB testing in patients on biologics who have previously tested negative and do not have new risk factors, as the American Rheumatology Society has recommended.^17^ Indeed, a recent study of repeat testing for latent TB in IBD patients showed the few new abnormal results were false positives.^18^ Another recent study found that indeterminate results were associated with delays in treatment and subsequent hospitalization.^19^ None of the patients with indeterminate results were diagnosed with latent TB.^19^ Professional GI societies in the United States might consider updating guidance of latent TB screening in IBD patients who previously tested negative and without new risk factors for exposure to avoid therapy interruption.

The major strength of our study is the use of a large dataset that captures patients in both academic and community settings, including information from inpatient and outpatient encounters as well as pharmacy data. One advantage of this claims data is the ability to capture complications even for patients readmitted to a different health system.

We attempted to limit the risk of miscoding by using a validated algorithm for IBD and requiring an antifungal prescription with a diagnosis code for fungal infection. Our dataset only included patients with commercial insurance in the United States, so the results may not be generalizable to patients with Medicare or Medicaid, the elderly, those without insurance, and to patients with IBD in non-US geographical locations where TB is endemic. While we are confident that medications administered as infusions were received because they have an associated Healthcare Common Procedure Coding System code, we cannot be certain that a patient was compliant in their usage of an injectable or oral medication prescribed for the treatment of IBD.

We used time-dependent variables to assess the association of medications with fungal infections. We chose a 90-day interval because many of the medications of interest are typically administered every 8 weeks. Some biologics are given more frequently than every 8 weeks, such as adalimumab, which can be given as often as every week. However, it is unclear how long these medications impact susceptibility to infection. They likely act longer than their last dose. Our estimates may be conservative because if a patient developed a fungal infection a few days into a subsequent 90-day interval, the association with the medication in the previous interval would not be reflected in the HR. We tested 30- and 60-day time intervals in sensitivity analysis and the HR for anti-TNFs and corticosteroids were similar.

## Conclusions

We found that invasive fungal infections were more common than TB in patients with IBD in a US commercial claims database. Anti-TNFs were associated with 1.6-fold increased risk of invasive fungal infections, but corticosteroids were associated with much higher risk (5.4-fold). Future studies should examine whether newer corticosteroid-sparing biologics such as vedolizumab and ustekinumab may be safer than anti-TNFs, particularly in patients at increased risk for fungal infections, such as those on TPN.

## Supplementary Material

otad010_suppl_Supplementary_Data

## Data Availability

SAS code used for analysis is available upon request. Access to marketscan is restricted by institutional and contractual obligations and cannot be requested.
