# Supplementary material for: Corticosteroids Increase the Risk of Invasive Fungal Infections More Than Tumor Necrosis Factor-Alpha Inhibitors in Patients With Inflammatory Bowel Disease
Source: Crohns Colitis 360. 2023 Feb 19;5(2):otad010. doi: 10.1093/crocol/otad010 (PMC9999356; doi:10.1093/crocol/otad010)
Supplement: otad010_suppl_Supplementary_Data [file otad010_suppl_supplementary_data.docx]

**Supplementary Data Content**

Table S1: Variables with ICD-9-CM codes, CPT-4 or ICD-9-CM procedure codes or drug codes

| **DESCRIPTION OF CONDITION, PROCEDURE, OR DRUG** | **ICD-9-CM/ICD-10-CM DIAGNOSIS CODES** | **CPT-4/HCPCS, ICD-9-CM, OR ICD-10-PCS**  **PROCEDURE CODES** | **Drug codes with HCPCS code if applicable** |
| --- | --- | --- | --- |
| Crohn’s disease | 555.x  K50.x |  |  |
| Ulcerative colitis | 556.x  K51.x |  |  |
| Crohn’s fistula | 537.4, 567.22, 567.21, 569.5, 569.81, 569.83, 593.82, 596.1, 619.1  K31.6, K65.0, K65.1, K63.0, K63.1, K63.2, N82, N32.1 |  |  |
| Crohn’s stricture | 560.1, 560.2, 560.30, 560.8, 560.81, 560.89, 568.0, 560.9, 537.3  K56.0, K56.1, K56.2, K56.49, K56.5, K56.6, K31.5 |  |  |
| IBD-related surgery |  | 45.81, 45.82, 45.83, 45.61–45.63, 45.71–45.79, 54.51, 46.72, 46.74, 46.76, 48.73, 48.93, 49.11, 49.12, 49.73, 70.72, 70.73, 70.74, 70.75, 49.01, 54.0, 54.19, 48.91, 46.80, 46.81, 46.82, 46.85, 48.75, 48.76, 96.22, 96.24, 54.51, 54.59, 46.40, 46.41, 45.61, 45.62, 45.63, 48.41, 48.49, 48.5, 48.61, 48.62, 48.63, 48.64, 48.65, 48.69, 46.01, 46.02, 46.03, 46.04, 46.10, 46.11, 46.13, 46.20, 46.21, 46.22, 46.23, 46.24, 46.31, 46.32, 46.39, 46.50, 46.51, 46.52  44140, 44141, 44143, 44144, 44145, 44146, 44147, 44150, 44151, 44155, 44156, 44157, 44158, 44160, 44204, 44205, 44206, 44207, 44208, 44210, 44211, 44212, 44188, 44206, 44208, 50810, 57307, 44125, 44130, 44227, 44186, 44187, 45136, 44130, 45110, 45111, 45112, 45113, 45114, 45116, 45119, 45123, 45136, 45150, 45395, 45397, 45990, 46700, 46706, 46710, 46712, 44120, 44121, 44125, 44126, 44127, 44128, 44130, 44202, 44203, 44152, 44153, 44602, 44603, 44604, 44605, 44700, 44701**,** 44799, 45500, 45505, 45550, 45562, 45563, 45905, 45910, 45915, 44005, 44180, 44200, 44615, 45150, 46700, 44312, 44314, 44340, 44345, 44346, 45120, 45121, 45123, 45126, 44201, 44300, 44310, 44316, 44320, 44322, 45805, 45825, 45820, 44620, 44625, 44626, 44640, 44650, 44660, 44661, 45800, 46715, 46716, 46020, 46030, 57300, 57305, 57308, 46270, 46275, 46280, 46285 |  |
| Opioids |  |  | Fentanyl, hydrocodone, hydromorphone, methadone, morphine, oxycodone, buprenorphine, codeine |
| TPN |  | S9364, S9365, S9366, S9367, S9368, B4164, B4168, B4172, B4176, B4178, B4180, B4185, B4189, B4193, B4197, B4199, B4216, B4220, B4222,  B4224, B5000, B5100, B5200 |  |
| Lymphoma | 200–202, 203.0, 238.6  C81–C88, C90.0, C90.2, C96 |  |  |
| Leukemia | 203.1x, 203.8x, 204–208  C91-C96 |  |  |
| Candidiasis, nonesophageal | B37.5 Candidal meningitis  B37.6 Candidal endocarditis  B37.7 Candidal sepsis  B37.84 Candidal otitis externa  112.5 Disseminated candidiasis  112.81 Candidal endocarditis  112.82 Candidal otitis externa  112.83 Candidal meningitis |  |  |
| Esophageal candidiasis | 112.84  B37.81 |  |  |
| Coccidiomycosis | 114  B38 |  |  |
| Histoplasmosis | 115  B39 |  |  |
| Blastomycosis | 116.0  B40 |  |  |
| Paracoccidiomycosis | 116.1  B41 |  |  |
| Aspergillosis | 117.3, 484.6  B44 |  |  |
| Cryptococcus | 117.5, 321.0  B45 |  |  |
| Mucormycosis | 117.7  B46 |  |  |
| *Pneumocystis jiroveci* | 136.3  B59 |  |  |
| Trimethoprim-sulfamethoxazole |  |  | Trimethoprim-sulfamethoxazole |
| Atovaquone |  |  | Atovaquone |
| Clindamycine + primaquine |  |  | Clindamycine + primaquine |
| Trimethoprim + dapsone |  |  | Trimethoprim + dapsone |
| Pentamidine |  |  | Pentamidine |
| Fungal meningitis | 321.1  G02.1 |  |  |
| Fungal pneumonia | 484.7  J17.2 |  |  |
| Amphotericin |  | J0285, J0287, J0288, J0289 | Amphotericin B deoxycholate  Amphotericin B lipid complex  Liposomal amphotericin B  IV |
| Fluconazole |  | J1450 | Fluconazole  PO or IV |
| Isavuconazole |  | J1833 | Isavuconazole  PO or IV |
| Itraconazole |  | J1835 | Itraconazole  PO or IV |
| Ketoconazole |  |  | Ketoconazole  PO or IV |
| Voriconazole |  | J3465 | Voriconazole  PO or IV |
| Posaconazole |  | J3490 | Posaconazole  PO or IV |
| Anidulafungin |  | J0348 | Anidulafungin  IV |
| Caspofungin |  | J0637 | Caspofungin  IV |
| Micafungin |  | J2248 | Micafungin  IV |
| Flucytosine |  |  | Flucytosine  PO or IV |
| Terbinafine |  |  | Terbinafine  PO |
| Sulfasalazine and derivatives including mesalamine, olsalazine, balsalazide. |  |  | Sulfasalazine, mesalamine, olsalazine, balsalazide |
| 6MP or azathioprine |  |  | Azathioprine (J7500, J7501), 6-mercaptopurine (S0108) |
| Methotrexate. Available PO, SQ or IM. We want all of them. |  | J9260, J9250 | Methotrexate  (PO, SQ or IM) |
| Infliximab |  | J1745 | Infliximab |
| Adalimumab | . | J0135 | Adalimumab |
| Certolizumab |  | J0717 | Certolizumab |
| Golimumab |  | J1602 | Golimumab |
| Vedolizumab |  | J3380 | Vedolizumab |
| Natalizumab |  | J2323 | Natalizumab |
| Ustekinumab |  | J3357 | Ustekinumab |
| Corticosteroids |  |  | Prednisone,  hydrocortisone, dexamethasone (PO) |
| Budesonide |  |  | Budesonide (PO) |
| Active tuberculosis | 010-0180  A15-A19 |  |  |
| Latent tuberculosis | 795.51, ICD9 795.52  R76.11, R76.12 |  |  |
| Isoniazid |  |  | Isoniazid |
| Rifampin |  |  | Rifampin |
| Rifapentine |  |  | Rifapentine |
| Rifabutin |  |  | Rifabutin |
| Ethambutol |  |  | Ethambutol |
| Pyrazinamide |  |  | Pyrazinamide |
| Moxifloxacin |  |  | Moxifloxacin |
| Diabetes, uncomplicated | 250.0-250.3, 648.0 |  |  |
| Anemia | 280.0, 648.2  280.1-281.9, 285.2, 285.9 |  |  |

Table S2: Hazard ratios from a Cox proportional hazards model for risk of fungal infections in patients with inflammatory bowel disease

|  | Hazard Ratio | 95% Lower | 95% Upper | P value |
| --- | --- | --- | --- | --- |
| Anti-TNF* | 1.631 | 1.291 | 2.061 | < 0.0001 |
| Corticosteroids* | 5.356 | 4.605 | 6.228 | < 0.0001 |
| TPN* | 16.286 | 11.357 | 23.354 | < 0.0001 |
| Female | 0.870 | 0.750 | 1.008 | 0.0638 |
| Diabetes mellitus | 1.527 | 1.125 | 2.071 | 0.0065 |
| Complicated Crohn’s** | 1.141 | 0.824 | 1.579 | 0.4275 |
| Disease location |  |  |  |  |
| Ulcerative proctitis | Ref |  |  |  |
| Ulcerative left-sided | 1.116 | 0.609 | 2.046 | 0.7223 |
| Pancolitis | 1.276 | 0.833 | 1.955 | 0.2620 |
| IBD surgery/admit/ED*** | 1.535 | 1.301 | 1.811 | < 0.0001 |
| Anemia | 2.096 | 1.634 | 2.690 | < 0.0001 |
| Opioids | 1.331 | 1.128 | 1.571 | 0.0007 |
| Leukemia | 10.171 | 5.576 | 18.553 | < 0.0001 |
| Lymphoma | 5.697 | 3.241 | 10.013 | < 0.0001 |
| Age |  |  |  |  |
| <18 | 1.177 | 0.756 | 1.833 | 0.4713 |
| 18-24 | Ref |  |  |  |
| 25-29 | 1.117 | 0.717 | 1.742 | 0.6245 |
| 30-34 | 1.272 | 0.844 | 1.916 | 0.2505 |
| 35-39 | 1.210 | 0.806 | 1.817 | 0.3573 |
| 40-44 | 1.421 | 0.965 | 2.093 | 0.0749 |
| 45-49 | 1.646 | 1.136 | 2.386 | 0.0084 |
| 50-54 | 1.527 | 1.056 | 2.207 | 0.0245 |
| 55-59 | 1.662 | 1.150 | 2.402 | 0.0069 |
| 60+ | 1.755 | 1.182 | 2.606 | 0.0053 |

Anti-TNF, anti-tumor necrosis factor-alpha inhibitors

IBD, inflammatory bowel disease

TPN, total parenteral nutrition

* Time-dependent variables

**Includes Crohn’s-related fistula or stricture. See Table S1 in Supplementary Digital Content for list of codes used.

***Includes IBD-related surgery, all cause ED in the baseline period. See Table S1 in Supplementary Digital Content for list of codes used.

Figure S1. Hazard ratios from a Cox proportional hazards model for risk of invasive fungal infections in patients with inflammatory bowel disease including immunomodulators

Anti-TNF, anti-tumor necrosis factor-alpha inhibitors

IM, immunomodulators

IBD, inflammatory bowel disease

TPN, total parenteral nutrition

Anti-TNF, corticosteroids and TPN were time-dependent variables. Model also adjusted for age. See Table S2 in Supplementary Data Content for hazard ratios for age groups.

*Includes IBD-related surgery, all-cause emergency department (ED) visits, and all-cause hospitalizations in the baseline period. See Table S1 in Supplementary Data Content for list of codes used.

**Includes Crohn’s-related fistula or stricture. See Table S1 in Supplementary Data Content for list of codes used.

Table S3: Hazard ratios from a Cox proportional hazards model for risk of fungal infections in patients with inflammatory bowel disease using a 30-day interval for time-dependent variables

|  | Hazard Ratio | 95% Lower | 95% Upper | P value |
| --- | --- | --- | --- | --- |
| Anti-TNF* | 1.622 | 1.264 | 2.082 | < 0.0001 |
| Corticosteroids* | 5.543 | 4.751 | 6.467 | < 0.0001 |
| TPN* | 14.919 | 9.778 | 22.765 | < 0.0001 |
| Female | 0.890 | 0.768 | 1.032 | 0.1222 |
| Diabetes mellitus | 1.504 | 1.109 | 2.041 | 0.0087 |
| Complicated Crohn’s** | 1.183 | 0.855 | 1.636 | 0.3117 |
| UC Disease location |  |  |  |  |
| Ulcerative proctitis | Ref |  |  |  |
| Ulcerative left-sided | 1.132 | 0.618 | 2.075 | 0.6883 |
| Pancolitis | 1.289 | 0.842 | 1.975 | 0.2429 |
| IBD surgery/admit/ED*** | 1.562 | 1.324 | 1.843 | < 0.0001 |
| Anemia | 2.155 | 1.681 | 2.764 | < 0.0001 |
| Opioids | 1.375 | 1.165 | 1.623 | 0.0002 |
| Leukemia | 10.349 | 5.678 | 18.862 | < 0.0001 |
| Lymphoma | 5.818 | 3.312 | 10.220 | < 0.0001 |
| Age |  |  |  |  |
| <18 | 1.182 | 0.759 | 1.841 | 0.4594 |
| 18-24 | Ref |  |  |  |
| 25-29 | 1.119 | 0.718 | 1.745 | 0.6197 |
| 30-34 | 1.273 | 0.845 | 1.917 | 0.2490 |
| 35-39 | 1.202 | 0.801 | 1.804 | 0.3750 |
| 40-44 | 1.404 | 0.953 | 2.067 | 0.0857 |
| 45-49 | 1.616 | 1.115 | 2.341 | 0.0113 |
| 50-54 | 1.483 | 1.027 | 2.146 | 0.0356 |
| 55-59 | 1.615 | 1.117 | 2.334 | 0.0108 |
| 60+ | 1.710 | 1.152 | 2.538 | 0.0078 |

Anti-TNF, anti-tumor necrosis factor-alpha inhibitors

IBD, inflammatory bowel disease

TPN, total parenteral nutrition

* Time-dependent variables

**Includes Crohn’s-related fistula or stricture. See Table S1 in Supplementary Digital Content for list of codes used.

*** Includes IBD-related surgery, all-cause emergency department (ED) visits, and all-cause hospitalizations in the baseline period. See Table S1 in Supplementary Digital Content for list of codes used.

Table S4 Hazard ratios from a Cox proportional hazards model for risk of fungal infections in patients with inflammatory bowel disease using a 60-day interval for time-dependent variables

|  | Hazard Ratio | 95% Lower | 95% Upper | P value |
| --- | --- | --- | --- | --- |
| Anti-TNF* | 1.650 | 1.300 | 2.095 | < 0.0001 |
| Corticosteroids* | 5.447 | 4.682 | 6.337 | < 0.0001 |
| TPN* | 17.091 | 11.792 | 24.772 | < 0.0001 |
| Female | 0.878 | 0.758 | 1.018 | 0.0858 |
| Diabetes mellitus | 1.517 | 1.118 | 2.058 | 0.0074 |
| Complicated Crohn’s** | 1.142 | 0.824 | 1.582 | 0.4245 |
| UC Disease location |  |  |  |  |
| Ulcerative proctitis | Ref |  |  |  |
| Ulcerative left-sided | 1.123 | 0.613 | 2.058 | 0.7082 |
| Pancolitis | 1.278 | 0.834 | 1.958 | 0.2593 |
| IBD surgery/admit/ED*** | 1.543 | 1.307 | 1.821 | < 0.0001 |
| Anemia | 2.103 | 1.639 | 2.699 | < 0.0001 |
| Opioids | 1.345 | 1.140 | 1.588 | 0.0005 |
| Leukemia | 10.378 | 5.698 | 18.902 | < 0.0001 |
| Lymphoma | 5.687 | 3.238 | 9.989 | < 0.0001 |
| Age |  |  |  |  |
| <18 | 1.172 | 0.752 | 1.825 | 0.4833 |
| 18-24 | Ref |  |  |  |
| 25-29 | 1.118 | 0.717 | 1.743 | 0.6235 |
| 30-34 | 1.273 | 0.845 | 1.917 | 0.2488 |
| 35-39 | 1.204 | 0.803 | 1.809 | 0.3674 |
| 40-44 | 1.413 | 0.960 | 2.080 | 0.0799 |
| 45-49 | 1.634 | 1.128 | 2.368 | 0.0095 |
| 50-54 | 1.509 | 1.044 | 2.182 | 0.0287 |
| 55-59 | 1.646 | 1.139 | 2.379 | 0.0080 |
| 60-64 | 1.740 | 1.172 | 2.583 | 0.0060 |

Anti-TNF, anti-tumor necrosis factor-alpha inhibitors

IBD, inflammatory bowel disease

TPN, total parenteral nutrition

* Time-dependent variables

**Includes Crohn’s-related fistula or stricture. See Table S1 in Supplementary Digital Content for list of codes used.

***Includes IBD-related surgery, all-cause emergency department (ED) visits, and all-cause hospitalizations in the baseline period. See Table S1 in Supplementary Digital Content for list of codes used.

Table S5: Hazard ratios from a Cox proportional hazards model for risk of fungal infections in patients with inflammatory bowel disease including only moderate or high dose steroids

|  | Hazard Ratio | 95% Lower | 95% Upper | P value |
| --- | --- | --- | --- | --- |
| Anti-TNF* | 1.760 | 1.392 | 2.225 | < 0.0001 |
| Corticosteroids* | 4.738 | 4.023 | 5.580 | < 0.0001 |
| TPN* | 17.146 | 11.931 | 24.638 | < 0.0001 |
| Female | 0.891 | 0.768 | 1.033 | 0.1264 |
| Diabetes mellitus | 1.514 | 1.116 | 2.054 | 0.0077 |
| Complicated Crohn’s** | 1.140 | 0.822 | 1.580 | 0.4330 |
| UC Disease location |  |  |  |  |
| Ulcerative proctitis | Ref |  |  |  |
| Ulcerative left-sided | 1.160 | 0.633 | 2.126 | 0.6310 |
| Pancolitis | 1.361 | 0.888 | 2.084 | 0.1569 |
| IBD surgery/admit/ED*** | 1.542 | 1.306 | 1.821 | < 0.0001 |
| Anemia | 2.172 | 1.690 | 2.792 | < 0.0001 |
| Opioids | 1.401 | 1.187 | 1.654 | < 0.0001 |
| Leukemia | 10.382 | 5.668 | 19.016 | < 0.0001 |
| Lymphoma | 6.019 | 3.411 | 10.623 | < 0.0001 |
| Age |  |  |  |  |
| <18 | 1.218 | 0.782 | 1.897 | 0.3835 |
| 18-24 | ref |  |  |  |
| 25-29 | 1.129 | 0.724 | 1.760 | 0.5930 |
| 30-34 | 1.288 | 0.855 | 1.940 | 0.2264 |
| 35-39 | 1.236 | 0.824 | 1.856 | 0.3058 |
| 40-44 | 1.457 | 0.990 | 2.146 | 0.0565 |
| 45-49 | 1.706 | 1.177 | 2.473 | 0.0048 |
| 50-54 | 1.585 | 1.096 | 2.292 | 0.0144 |
| 55-59 | 1.743 | 1.205 | 2.520 | 0.0032 |
| 60-64 | 1.850 | 1.252 | 2.761 | 0.0021 |

Anti-TNF, anti-tumor necrosis factor-alpha inhibitors

IBD, inflammatory bowel disease

TPN, total parenteral nutrition

* Time-dependent variables

**Includes Crohn’s-related fistula or stricture. See Table S1 in Supplementary Digital Content for list of codes used.

*** Includes IBD-related surgery, all-cause emergency department (ED) visits, and all-cause hospitalizations in the baseline period. See Table S1 in Supplementary Digital Content for list of codes used.

Table S6: Hazard ratios from a Cox proportional hazards model for risk of fungal infections including esophageal candidiasis in patients with inflammatory bowel disease

|  | Hazard Ratio | 95% Lower | 95% Upper | P value |
| --- | --- | --- | --- | --- |
| Anti-TNF* | 2.236 | 1.994 | 2.508 | <0.0001 |
| Corticosteroids* | 5.384 | 4.958 | 5.847 | <0.0001 |
| TPN* | 8.884 | 6.899 | 11.440 | <0.0001 |
| Female | 1.101 | 1.014 | 1.194 | 0.0217 |
| Diabetes mellitus | 1.504 | 1.267 | 1.785 | <0.0001 |
| Complicated Crohn’s** | 0.851 | 0.697 | 1.039 | 0.1127 |
| UC Disease location |  |  |  |  |
| Ulcerative proctitis | Ref |  |  |  |
| Ulcerative left-sided | 1.050 | 0.741 | 1.489 | 0.7830 |
| Pancolitis | 1.350 | 1.059 | 1.719 | 0.0152 |
| IBD surgery/admit/ED*** | 1.655 | 1.515 | 1.809 | <0.0001 |
| Anemia | 1.707 | 1.476 | 1.974 | <0.0001 |
| Opioids | 1.623 | 1.486 | 1.774 | <0.0001 |
| Leukemia | 4.263 | 2.527 | 7.191 | <0.0001 |
| Lymphoma | 3.400 | 2.203 | 5.249 | <0.0001 |
| Age |  |  |  |  |
| <18 | 1.468 | 1.181 | 1.826 | 0.0006 |
| 18-24 | Ref |  |  |  |
| 25-29 | 0.933 | 0.739 | 1.178 | 0.5604 |
| 30-34 | 1.106 | 0.893 | 1.369 | 0.3555 |
| 35-39 | 1.080 | 0.875 | 1.332 | 0.4736 |
| 40-44 | 1.276 | 1.045 | 1.558 | 0.0170 |
| 45-49 | 1.418 | 1.170 | 1.720 | 0.0004 |
| 50-54 | 1.246 | 1.027 | 1.511 | 0.0260 |
| 55-59 | 1.469 | 1.213 | 1.780 | <0.0001 |
| 60+ | 1.562 | 1.270 | 1.921 | <0.0001 |

Anti-TNF, anti-tumor necrosis factor-alpha inhibitors

IBD, inflammatory bowel disease

TPN, total parenteral nutrition

* Time-dependent variables

**Includes Crohn’s-related fistula or stricture. See Table S1 in Supplementary Digital Content for list of codes used.

***Includes IBD-related surgery, all cause ED in the baseline period. See Table S1 in Supplementary Digital Content for list of codes used.
